# Characterization of a novel *Achromobacter xylosoxidans* specific siphoviruse: phiAxp-1

**DOI:** 10.1038/srep21943

**Published:** 2016-02-24

**Authors:** Erna Li, Jiangtao Zhao, Yanyan Ma, Xiao Wei, Huan Li, Weishi Lin, Xuesong Wang, Chao Li, Zhiqiang Shen, Ruixiang Zhao, Aimin Jiang, Huiying Yang, Jing Yuan, Xiangna Zhao

**Affiliations:** 1 College of Food Science, South China Agricultural University, Guangzhou, China, 510642; 2 Emergency Department, the Fifth Affiliated Hospital of Zhengzhou University, Zhengzhou, China, 450052; 3 College of Food Science, Henan Institute of Science and Technology, Xinxiang, China, 453003; 4 Institute of Disease Control and Prevention, Academy of Military Medical Sciences, Beijing, China, 100071; 5 Key Laboratory of Risk Assessment and Control for Environment and Food Safety, Tianjin Institute of Health and Environmental Medicine, Tianjin, China, 300050; 6 State Key Laboratory of Pathogen and Biosecurity, Beijing Institute of Microbiology and Epidemiology, Beijing, China, 100071

## Abstract

Bacteriophages have recently been considered as an alternative biocontrol tool because of the widespread occurrence of antimicrobial-resistant *Achromobacter xylosoxidans*. Herein, we isolated a virulent bacteriophage (phiAxp-1) from a water sample of the Bohai sea of China that specifically infects *A. xylosoxidans*. Transmission electron microscopy revealed that phage phiAxp-1 belongs to the *Siphoviridae*. We sequenced the genome of phiAxp-1, which comprises 45,045 bp with 64 open reading frames. Most of the proteins encoded by phiAxp-1 have no similarity to sequences in the public databases. Twenty-one proteins with assigned functions share weak homology with those of other dsDNA bacteriophages infecting diverse hosts, such as *Burkholderia* phage KL1, *Pseudomonas* phage 73, *Pseudomonas* phage vB_Pae-Kakheti25, *Pseudomonas* phage vB_PaeS_SCH_Ab26, *Acinetobacter* phage IME_AB3 and *Achromobacter* phage JWX. The genome can be divided into different clusters for the head and tail structure, DNA replication and *mazG*. The sequence and genomic organization of bacteriophage phiAxp-1 are clearly distinct from other known *Siphoviridae* phages; therefore, we propose that it is a member of a novel genus of the *Siphoviridae* family. Furthermore, one-step growth curve and stability studies of the phage were performed, and the specific receptor of phiAxp-1 was identified as the lipopolysaccharide of *A. xylosoxidans*.

The worldwide rise of antibiotic-resistant bacterial strains has created the need for alternative means of controlling pathogenic bacteria^1^. Recently, interest in bacteriophages has increased because of their potential use as typing, diagnostic, therapeutic, decontaminating and bio-control agents^2^,^3^. In this study, *Achromobacter xylosoxidans* phage phiAxp-1 was isolated and characterized biologically. *A. xylosoxidans* is an aerobic Gram-negative bacillus that is an opportunistic human pathogen of immunocompromised hosts^4^. As a motile bacterium from the *Achromobacter* genus of the *Alcaligenaceae* family^5^, it is mainly found in moist soil and different water sources^6^. Recently, *A. xylosoxidans* was recognized as an emerging nosocomial pathogen^7^. This bacterium is commonly associated with a range of respiratory infections^8^. Although a large diversity of phages targeting a broad range of *A. xylosoxidans* strains have been described^6^, only two phages, JWAlpha and JWDelta, have been studied in detail via whole genome sequencing^7^. Our research aimed to isolate and characterize novel *Achromobacter* phages to expand the repertoire of phages available for targeting clinically significant *A. xylosoxidans*. Genomic analysis of bacteriophages is an important preliminary step in the development of a phage therapy protocol^9^. In this manuscript, we sequenced the genome of phage phiAxp-1 and characterized it further by transmission electron microscopy (TEM), and one-step growth curve and stability studies. In addition, the host component bound by the phage was identified.

## Results and Discussion

### Morphology

**Figure 1:**
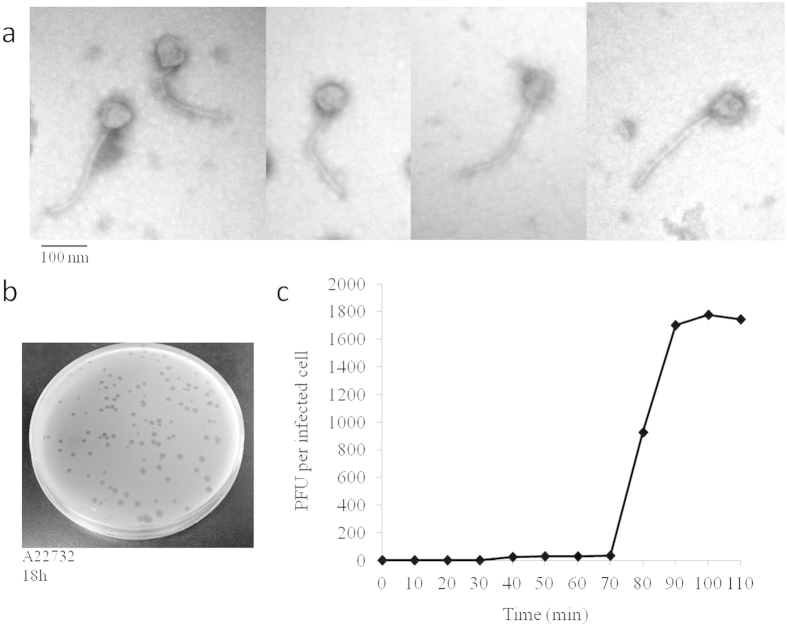
Isolated Achromobacter phage phiAxp-1. (**a**) Morphology of the bacteriophage. Transmission electron micrograph (TEM) of phage phiAxp-1 at ×120000. The phages were negatively stained with 2% uranyl-acetate. The bar indicates 100 nm. (**b**) Plaques of phage phiAxp-1 between 2 and 3 mm in diameter on *A. xylosoxidans* strain A22732. (**c**) One-step growth curve of the bacteriophage. Phages were grown in an exponential phase culture of *A. xylosoxidans* strain A22732. Shown are the PFU per infected cell in cultures at different time points. Each data point is the mean from three experiments.

Phage phiAxp-1 was purified successfully from a lysed lawn of the host bacteria *A. xylosoxidans* A22732. *A. xylosoxidans* strain A22732 harbours a conjugative imipenem-encoding plasmid and is resistant to multiple β-lactam antibiotics, including imipenem and meropenem^5^. Purified phages were negatively stained and examined by TEM (Fig. 1a). The head is approximately 66 nm in diameter. The phage particles are each associated with a long non-contractile tail of approximately 230 nm in length and 15 nm in width. Collectively, these morphological features indicated that this virus belongs to the family *Siphoviridae*. The phage produced large, clear, round plaques of 2–3 mm in diameter on a lawn of *A. xylosoxidans* A22732 (Fig. 1b). Multiplication parameters of phage phiAxp-1 were determined under one-step growth curve conditions (Fig. 1c). The latent period, defined as the time interval between adsorption and the beginning of the first burst, was about 75 min. The burst time of 90 min and the average burst size of 1,742 pfu/cell were determined, calculated as the ratio of the final count of liberated phage particles to the initial count of infected bacterial cells during the latent period. Host range tests suggested that phiAxp-1 was virulent specifically to only five strains of *A. xylosoxidans* strains among all species tested (n = 57) (Table 1). Besides the reported multidrug-resistant strain A22732, all the other four clinical *A. xylosoxidans* strains were determined to be resistant to aztreonam and tobramycin.

### General features of the phiAxp-1 genome

**Figure 2:**
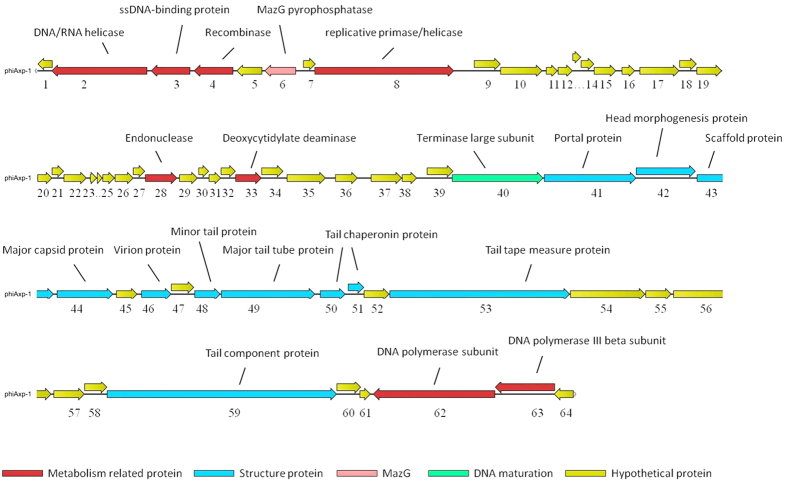
Genomic map of phiAxp-1. The genome map of phiAxp-1 was drawn using CLC Main Workbench, version 6.1.1 (CLC bio, Aarhus, Denmark). The bacteriophage phiAxp-1 genome is presented schematically with predicted open reading frames (ORFs) indicated by arrows; the direction of the arrow represents the direction of transcription.

**Figure 3:**
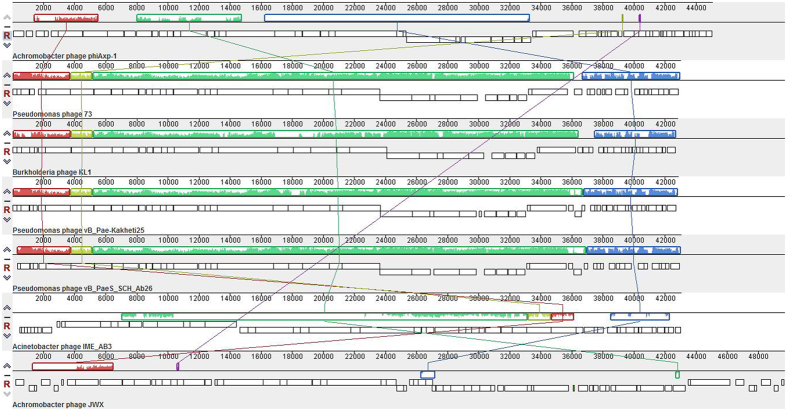
Multiple genome alignment performed using Mauve software (http://asap.ahabs.wisc.edu/mauve/) and the chromosomes of related phages. Similarity is represented by the height of the bars, which correspond to the average level of conservation in that region of the genome sequence. Completely white regions represent fragments that were not aligned or contained sequence elements specific to a particular genome. Boxes with identical colours represent local collinear blocks (LCB), indicating homologous DNA regions shared by two or more chromosomes without sequence rearrangements. LCBs indicated below the horizontal black line represent the reverse complements of the reference LCB.

The DNA sequence of phage phiAxp-1 is a circular double stranded DNA of 45,045 bp with a GC content of 56.02%. The genome contains 64 putative open reading frames (ORFs); however, only 21 ORFs share similarity at the protein level with other sequences deposited in GenBank. The predicted phiAxp-1 ORFs are between 30 and 1331 codons in length. Fifty-five (86%) of the phiAxp-1 genes are transcribed in one direction, designated as rightward on the genome map (Fig. 2), and nine genes are transcribed in the leftward direction. The GC content is approximately the same for both sets of ORFs. Fifty-eight of the 64 predicted ORFs start with an ATG codon, four ORFs use TTG, and two use GTG. At the ends of phiAxp-1 genes, there are 30 TAA stop codons, 30 TGA codons, three TAG codons and one AAA codon. Although we propose that phiAxp-1 is a siphovirus, the proteins of the phage have largely uncharacterized functions. Putative functional gene assignments included a large terminase subunit, capsid and tail genes, and DNA replication/modification/salvage genes. The phiAxp-1 genome has a modular organization, which is common among phages^10^. The proteins encoded by phiAxp-1 belong to four different functional categories: virion morphogenesis (including capsid and tail morphogenesis), DNA packaging, DNA replication, and MazG (a pyrophosphohydrolase^11^). Putative functional assignments and significant similarities to other sequences are listed in Table 2. The proteins with assigned functions share weak homology with proteins found in other dsDNA bacteriophages, infecting diverse hosts, such as *Burkholderia* phage KL1, *Pseudomonas* phage 73, *Pseudomonas* phage vB_Pae-Kakheti25, *Pseudomonas* phage vB_PaeS_SCH_Ab26, *Acinetobacter* phage IME_AB3 and *Achromobacter* phage JWX, with percent identities of 24–70%. Multiple genome alignments showed the weak homology of phiAxp-1 with these phages at the whole genome level (Fig. 3). In addition, 18 virion proteins were detected using liquid chromatography-electrospray ionization-tandem mass spectrometry (LC-ESI-MS/MS), of which 12 have been assigned putative functions (Table 3).

**Figure 4:**
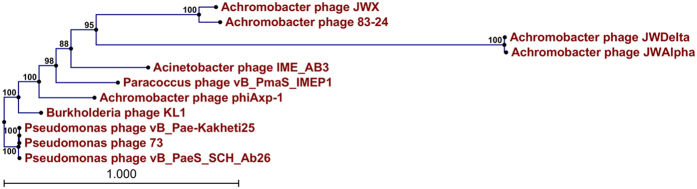
Phylogenetic tree based on large terminase subunits of selected bacteriophages. The large terminase subunits were compared using the ClustalW program, and the phylogenetic tree was generated using the neighbour-joining method and 1000 bootstrap replicates.

Of the 21 phiAxp-1 proteins with a suggested function, half of them are structural and morphogenesis proteins. Based on BLASTP analysis, the capsid morphogenesis and DNA packaging module contains genes encoding the large terminase subunit, capsid and tail proteins (Fig. 2). As a phage with *Siphoviral* morphotypes, the genes for head and tail assembly are arranged together, with the head genes 5′ to the tail genes^12^. Terminase genes are involved in initiation of DNA synthesis and are responsible for the packaging of the concatameric DNA in phage capsids^13^. *gp40* (terminase large subunit) shares similarity with the terminase large subunit protein from KL1 (70% identity), vB_PaeS_SCH_Ab26 (66% identity) and 73 (67% identity). Phylogenetic analysis of the available bacteriophage terminase proteins also demonstrated that phiAxp-1 could not be assigned to a branch (Fig. 4). Portal proteins are responsible for forming a ring that enables DNA to pass into the major capsid during assembly and out during infection, serving as a junction between the capsid and tail proteins^13^,^14^. *gp41* encodes a protein similar to the portal proteins found in the genomes of vB_Pae-Kakheti25 (48% identity), vB_PaeS_SCH_Ab26 (48% identity) and 73 (48% identity). *gp42* (head morphogenesis protein) shares weaker identity with the head morphogenesis protein from KL1 (28% identity). *gp43* (scaffold protein) is similar to vB_PaeS_SCH_Ab26 orf9 (46% identity), and *gp44* (major capsid protein) is similar to the major capsid protein of IME_AB3 (54% identity). The phiAxp-1 genome encodes six putative tail proteins, including a minor tail protein (*gp48*), a major tail tube protein (*gp49*), two tail chaperonin proteins (*gp50–51*), a tail tape measure protein (*gp53*) and a tail component protein (*gp59*) (Table 2). The tail tape measure protein (1044 amino acids [aa], encoded by *gp53*) is similar to the predicted KL1 tape measure protein orf21 (41% identity). The tape measure protein is important for the assembly of phage tails and is involved in tail length determination^15^,^16^. Importantly, the largest protein encoded by phiAxp-1 is the tail component protein (*gp59*, 1331 aa) rather than the tail tape measure protein, which is commonly the largest protein of siphovirus^12^. In addition, gp57–59 are similar to JWX orf25–27 (42–50% identity).

phiAxp-1 encodes a cluster of proteins involved in DNA replication. These DNA- or nucleotide binding proteins include DNA polymerase (*gp62*), DNA polymerase III β subunit (*gp63*), DNA/RNA helicase (*gp2*), single-stranded DNA-binding protein (*gp3*), recombinase (*gp4*), replicative primase/helicase (*gp8*) and exonuclease (*gp28*) (Fig. 2). *gp62* (DNA polymerase) and *gp63* (DNA polymerase III β subunit) are similar to those of IME_AB3 (55% identity) and vB_PmaS_IMEP1 (33% identity), respectively, and are thought to be responsible for genome replication. *gp33* (deoxycytidylate deaminase) is similar to *Thermus* phage TMA orf38 (46% identity) and is involved in nucleotide salvage. A notable protein encoded by phiAxp-1 is MazG, which is a pyrophosphohydrolase that acts on ppGpp, one of the signalling molecules produced by bacteria during the stringent response^9^. The *mazG* gene provides a significant selective advantage to phages^17^. The putative MazG protein is encoded by phiAxp-1 *gp6* and contains a MazG nucleotide pyrophosphohydrolase domain and is similar to the putative MazG protein from phage IME_AB3 (37% identity).

### Stability studies

**Figure 5:**
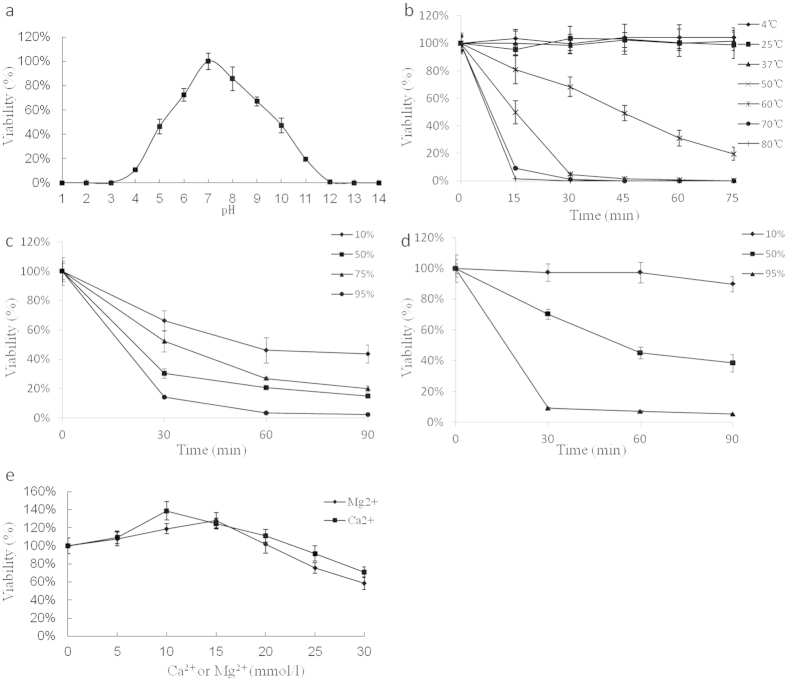
Stability of phage phiAxp-1 under different pHs (**a**), temperatures (**b**), ethanol concentrations (**c**), isopropanol concentrations (**d**) and divalent ion concentrations (**e**). The results were expressed as a percentage of the initial viral counts. Each assay was performed as three repetitions and the values represented are the means.

Stability studies of phage phiAxp-1 were conducted with different pHs, disinfectants, temperatures and ions, using a temperature-controlled incubator or water baths. The results are summarized in Fig. 5. The phage was most stable at pH 7, there was a significant reduction in the phage titre either above or below pH 7; the phage titre was further decreased under extremely acidic (pH 4) or basic (pH 12) conditions (Fig. 5a). No significant loss of phage titre was observed from 4 to 37  °C. However, the phage titre dramatically decreased when the temperature is over 50 °C (Fig. 5b). The activities of phage phiAxp-1 were affected in the presence of ethanol (Fig. 5c), the phage was resistant to isopropanol (Fig. 5d) at low concentrations (10%, v/v), whereas it became unstable with increasing concentrations: there was a significant reduction in phage titre at high concentration (95%, v/v). Many phages require divalent ions such as Ca^2+^ or Mg^2+^ for attachment or intracellular growth^18^. It may be necessary to treat phages with Ca^2+^ or Mg^2+^ to obtain an efficient phage infection. The effects of divalent ions on phage amplification were evaluated and the phage revealed divalent cation dependency for optimal infectivity. Divalent cations at no more than 20 mM were beneficial for plaque development (Fig. 5e).

### Identification of the phage receptor

**Figure 6:**
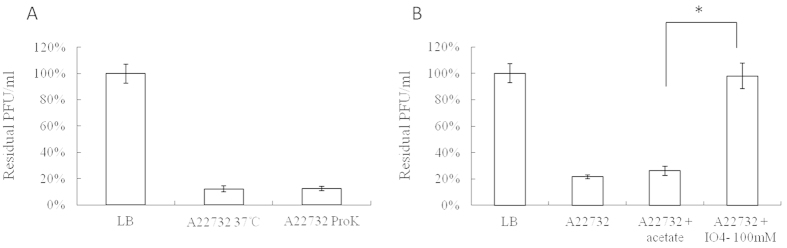
Effects of different treatments of bacteria on phiAxp-1 adsorption. The results are shown as residual PFU percentages. (**A**) Effect of proteinase K treatment on the adsorption of phiAxp-1 to *A. xylosoxidans* strain A22732. (**B**) Effect of periodate treatment on the adsorption of phiAxp-1 to *A. xylosoxidans* strain A22732. The control (LB and “A22732 + acetate”), untreated strain (A22732), and treatment (“A22732 + ProtK” for proteinase K treatment and “A22732 + IO^4−^” for periodate treatment) groups were tested for adsorption, as indicated in the *x-*axes. Error bars denote statistical variations. Significance was determined by a Student’s *t* test for comparison between the treated and the untreated groups. *P 0.05.

**Figure 7:**
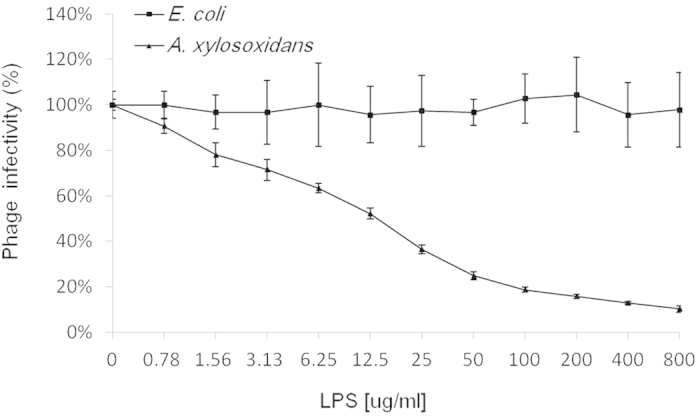
Inactivation of phiAxp-1 by lipopolysaccharide (LPS) derived from *A. xylosoxidans* A22732. % Infectivity was determined after 1 hr of incubation at 37 °C. Error bars denote statistical variations.

In Gram-negative bacteria, outer membrane proteins or lipopolysaccharide (LPS) may function as specific phage receptors^19^. Therefore, it was necessary to test whether the degradation of cell surface proteins or LPS could inhibit phiAxp-1 binding^20^. *A. xylosoxidans* cells were treated with either proteinase K (to destroy surface proteins) or periodate (to destroy surface carbohydrates) before the phage adsorption assay to determine the possible nature of the phage receptor^20^. phiAxp-1 exhibited high infection efficiency when mixed with untreated and proteinase K-treated *A. xylosoxidans* cells (Fig. 6a): the majority of the phages were removed from the suspension after centrifugation by binding to *A. xylosoxidans* cells. This suggested that the functional receptor is not a protein. The broad substrate specificity of proteinase K meant that the possibility that the receptor is a protein resistant to proteinase K is unlikely^19^. When the phage was incubated with periodate-treated *A. xylosoxidans*, the majority of the phages remained in the supernatant (Fig. 6b). The significant increase of free phage particles suggested that the phages were unable to efficiently adsorb onto the periodate-treated bacteria. Therefore, the *A. xylosoxidans* receptor recognized and bound by phiAxp-1 is a carbohydrate structure, most likely LPS. Significant inactivation of phages was further confirmed using LPS purified from *A. xylosoxidans*, which demonstrated LPS is the adsorption target (receptor) of this phage (Fig. 7). The results revealed direct correlation between *A. xylosoxidans* LPS concentration and phage infectivity inhibition, and 12.5 μg/ml of LPS was sufficient to inhibit the binding activity of 50% of 4.7 × 10^4^ pfu phiAxp-1. LPS of *Escherichia coli* 0111:B4 was used as a negative control. As shown in Fig. 7, *E. coli* LPS showed no phage inactivating ability compared with that from *A. xylosoxidans* LPS, thus indicating that LPS from *A. xylosoxidans* is specific for phage phiAxp-1. In this respect, it is consistent with the features of most phages with Gram-negative bacterial hosts.

## Concluding Remarks

The clinical relevance of nosocomially-acquired infections caused by multi-resistant *Achromobacter* strains is increasing rapidly, becoming a critical problem^7^. Phages are re-emerging as promising potential therapies for the treatment of bacterial infections^21^. Here, we report a preliminary analysis of *A. xylosoxidans* bacteriophage phiAxp-1. This article presents the sequence analysis and a detailed genome annotation of phage phiAxp-1. The genomic data constitute an important resource to study and engineer phages to control specific bacterial species^22^. The analysis showed that phiAxp-1 does not easily fit into previously established groups of dsDNA bacterial viruses and may represent a distinct branch of the *Siphoviridae* family.

Stability is the primary requirement for any possible commercial use of the phage, which can reduce the cost of storage significantly^23^. Therefore, in this study, the stability tests on phage phiAxp-1 under different pHs, temperatures, disinfectants and ions were performed for the potential practical application of phiAxp-1. Despite its importance, the molecular interactions between phiAxp-1 and the surface of *A. xylosoxidans* are still poorly understood. Phages bind to unique host-specific structures, allowing them to recognize a suitable host in a mixed bacterial population^24^. In this study, periodate treatment of *A. xylosoxidans*, but not proteinase K treatment, inhibited phage binding. Furthermore, purified LPS from the *A. xylosoxidans* showed phage-inactivating capacities thus confirmed that LPS of *A. xylosoxidans* is the receptor of phage phiAxp-1.

The emergence of phage-resistant mutants affecting phage receptors is a major concern regarding the use of phage therapy^25^. The LPS of Gram-negative bacteria commonly represents an important virulence factor and is of great significance in the pathophysiology of many disease processes^26^. Thus, the phage-resistant mutants resulting from the loss or alteration of the receptor will be avirulent or attenuated. Such mutants do not pose a problem during bacteriophage treatment^25^. Our future work will explore this possibility. These results suggest that phage phiAxp-1 is a promising candidate for controlling *A. xylosoxidans* and represents an advance in our current knowledge of *A. xylosoxidans* phages.

## Methods

### Bacterial strains and growth media

Luria-Bertani (LB) broth medium was used to grow the bacterial strains and to propagate the phage. *A. xylosoxidans* strain A22732 was used as the indicator strain for phage isolation.

### Isolation of phage and host range determination

phiAxp-1 was isolated from a water sample of the Bohai sea of China using a double agar overlay plaque assay, as described previously for the isolation of lytic phages^27^. The water sample was centrifuged at 8,000 × *g* for 10 min to remove the solid impurities. The supernatants were filtered through a 0.22-μm pore-size membrane filter to remove bacterial debris. The filtrates were then mixed with *A. xylosoxidans* culture to enrich the phage at 37 °C. The culture was centrifuged, and the supernatant was filtered through a 0.22-μm pore-size membrane to remove the residual bacterial cells. Aliquots of the diluted filtrate were mixed with *A. xylosoxidans* culture. Then, 3 mL of molten top soft nutrient agar (0.7% agar) were overlaid on the solidified base nutrient agar (1.5% agar)^28^. Following incubation for 10 h at 37 °C, clear phage plaques were picked from the plate. The phage titre was determined using the double-layered method. The host range of the phage was tested against 57 clinical strains from our microorganism centre, as determined by standard spot tests^29^. Briefly, 10 μl from a purified phage suspension containing approximately 10^8^ pfu/mL were spotted in the middle of a lawn of bacteria and left to dry before incubation overnight. Bacterial sensitivity to a bacteriophage was established by bacterial lysis at the spot where the phage was deposited. Each strain was tested three times at 37 °C.

### TEM

To prepare phiAxp-1 for transmission electron microscopy studies, cell debris from 500 mL of *A. xylosoxidans* strain A22732 infected with phiAxp-1 was pelleted by centrifugation. Phage particles were precipitated with 1 M NaCl and 10% polyethylene glycol (PEG) 8000 at 4 °C with stirring for 60 min. The precipitated phage particles were harvested. Phage particles were resuspended in Saline - magnesium (SM) diluent plus gelatin (SMG) (50 mM Tris-HCl [pH 7.5] containing 100 mM NaCl, 8.1 mM MgSO_4_ and 0.01% (w/v) gelatin) and extracted with an equal volume of chloroform. After low-speed centrifugation, the aqueous phase was sedimented at about 25,000 × g for 60 min. Phage particles were negatively stained with 2% (wt/vol) phosphotungstic acid (pH 7), air dried, and examined under a Philips EM 300 electron microscope operated at 80 kV and 120 KEv.

### One-step growth curve

One-step growth experiments were performed as described previously^30^. Host strain *A. xylosoxidans* strain A22732 cells were harvested at exponential growth and resuspended in LB. The phage phiAxp-1 was added at a multiplicity of infection (MOI) of 0.0005 and allowed to adsorb for 5 min at room temperature. The mixture was centrifuged and the pellets containing infected cells were suspended in 10 ml of LB, followed by incubation at 37 °C. Samples were taken at 10 min intervals (up to 110 min) and immediately diluted, and then titres were determined by the double-layered-agar plate method.

### Stability studies

A temperature-controlled incubator or water bath was used to determine the stabilities at different pH, disinfectants, ions or temperatures. After the desired treatment, the tube was cooled down slowly, placed in an ice water bath and samples were assayed to determine surviving pfu. Briefly, for thermal stability tests, aliquots of phage suspensions were incubated at 37 °C (pH 1–14) for 60 min, and then the phage titres were tested by the double-layer agar method. For pH stability tests, aliquots of phage suspensions were incubated at 4, 25, 37, 50, 60, 70 and 80 °C for 75 min; the phage titres were determined every 15 min. For disinfectant sensitivity, aliquots of phage suspensions were incubated with different concentrations of ethanol or isopropanol for 90 min; the phage titres were determined every 30 min. For divalent ion sensitivity, aliquots of phage suspensions were assayed on solidified base nutrient agar (1.5% agar) with different concentrations of Mg^2+^ or Ca^2+^ and the phage titres were tested.

### Isolation of phage DNA, genome sequencing and assembly

phiAxp-1 DNA was extracted from purified phage particles with phenol-chloroform (24:1, vol/vol) and precipitated with 100% ethanol. The samples were visualized on 0.7–1.0% agarose gels, and the purified phage DNA was sequenced using an Illumina HiSeq2500 sequencer. The sequence reads were filtered to remove low quality sequences, trimmed to remove adaptor sequences and the filtered sequences were assembled. The final assembled sequence was searched against the current protein and nucleotide databases ( http://www.ncbi.nlm.nih.gov/) using Basic Local Alignment Search Tool (BLAST) software^31^. BLASTP was used to determine the similarity to described proteins in the National Center for Biotechnology Information [NCBI] database ( http://www.ncbi.nlm.nih.gov). The CLC Main Workbench, version 6.1.1 (CLC bio, Aarhus, Denmark) was used for genome annotation. Computer-based predictions were checked manually. Phylogenetic analysis with the published genome sequences of related phages was conducted using ClustalW (Slow/Accurate, IUB). Whole genome comparisons were carried out using Mauve^32^. LC/ESI/MS/MS spectra (Q-TOF Ultima API, Micromass UK Ltd.) were used to identify the phage proteins, as described previously^33^.

### Identification of the phage receptor

Receptor properties of phiAxp-1 were determined as described previously^19^. Briefly, *A. xylosoxidans* A22732 cultures were treated with sodium acetate (50 mM, pH 5.2) containing 100 mM IO^4−^ at room temperature for 2 h (protected from light) or proteinase K (0.2 mg/ml; Promega) at 37 °C for 3 h to determine whether proteinase K or periodate can destroy the phage receptor. The phage adsorption assay was then performed as previously described^20^. LB was used as a non-adsorbing control in each assay, and the phage titre in the control supernatant was set to 100%. Each assay was performed in duplicate and repeated twice^19^.

### Phage inactivation by LPS

LPS extraction from *A. xylosoxidans* was performed using an LPS extraction kit from Intron Biotechnology (17144; Boca Scientific, Boca Raton, FL, USA), according to the manufacturer’s instructions. LPS from *Escherichia coli* O111:B4 purchased from Sigma-Aldrich, Inc. (L2630; Sigma, USA) was used as a negative control to ensure that the possible effect was specific to *A. xylosoxidans* LPS. Both LPS of *Escherichia coli* O111:B4 and *A. xylosoxidans* A22732 are smooth type. The phage inactivation by LPS was performed as previously described^34^.

#### Nucleotide sequence accession number

The annotated genome sequence for the phage phiAxp-1 was deposited in the NCBI nucleotide database under the accession number KP313532.

## Additional Information

**How to cite this article**: Li, E. *et al.* Characterization of a novel *Achromobacter xylosoxidans* specific siphoviruse: phiAxp-1. *Sci. Rep.* **6**, 21943; doi: 10.1038/srep21943 (2016).

## Figures and Tables

**Table 1:** Host range infection of the phage phiAxp-1.

Species	ID	Infection
*Achromobacter xylosoxidans*	A22732	+
*A. xylosoxidans*	5271	+
*A. xylosoxidans*	844	+
*A. xylosoxidans*	201308881	+
*A. xylosoxidans*	6065	+
*Enterobacter aerogenes*	3-SP	−
*E. aerogenes*	201316724	−
*E. aerogenes*	2015–301	−
*E. aerogenes*	13208	−
*E. aerogenes*	A29864	−
*E. aerogenes*	A36179	−
*E. aerogenes*	AH10	−
*E. aerogenes*	AH12	−
*E. aerogenes*	AH13	−
*E. aerogenes*	AH14	−
*E. aerogenes*	AH15	−
*E. aerogenes*	AH17	−
*E. aerogenes*	AH18	−
*E. aerogenes*	AH2	−
*E. aerogenes*	AH20	−
*E. aerogenes*	AH21	−
*E. aerogenes*	AH22	−
*E. aerogenes*	AH24	−
*E. aerogenes*	AH25	−
*E. aerogenes*	AH28	−
*E. aerogenes*	AH29	−
*E. aerogenes*	AH3	−
*E. aerogenes*	AH30	−
*E. aerogenes*	AH32	−
*E. aerogenes*	AH33	−
*E. aerogenes*	AH34	−
*E. aerogenes*	AH36	−
*Escherichia coli*	ATCC 25922	−
*E. coli*	DH10B	−
*E. coli*	EC600	−
*Klebsiella pneumoniae*	ATCC BAA-1706	−
*K. pneumoniae*	ATCC BAA-2146	−
*K. pneumoniae*	ATCC BAA-1705	−
*K. pneumoniae*	K2044	−
*K. pneumoniae*	511	−
*Serratia marcescens*	wk2050	−
*S. marcescens*	201315732	−
*S. marcescens*	wj-1	−
*S. marcescens*	wj-2	−
*S. marcescens*	wj-3	−
*E. cloacae*	T5282	−
*E. cloacae*	TI3	−
*E. sakazakii*	45401	−
*E. sakazakii*	45402	−
*Leclercia adcarboxglata*	P10164	−
*Raoultella ornithinolytica*	YNKP001	−
*Stenotrophomonas maltophilia*	9665	−
*Citrobacter freundii*	P10159	−
*Vibrio parahaemolyticus*	J5421	−
*Pseudomonas aeruginosa*	PA01	−
*Acinetobacter baumannii*	N1	−
*Shigella sonnei*	#1083	−

−, absent; +, present.

**Table 2:** *Achromobacter* phage phiAxp-1 gene annotations.

Orfs	Nucleotide position	Strand	Length (aa)^a^	Mass (Da)	pI	Best match (aa identity)	GenBank accession number	E value	Conserved Protein Domain Family	Function
orf01	9–266	−	85	9653	7.80					
orf02	250–1911	−	553	61431	8.50	IME_AB3 gp17 (39%)	NC_023590	1e–128	cd00046; pfam00270; cd00079; smart00490; COG1061; smart00487; PRK13766	DNA/RNA helicase protein
orf03	1979–2659	−	226	23634	6.76	IME_AB3 gp15 (27%)	NC_023590	2e–09		ssDNA-binding protein
orf04	2726–3406	−	226	25248	8.68	KL1 orf33 (50%)	NC_018278	7e–67		recombinase
orf05	3467–3910	−	147	16336	7.30	IME_AB3 gp13 (32%)	NC_023590	2e–15		
orf06	3954–4496	−	180	19897	5.44	IME_AB3 gp12 (37%)	NC_023590	2e–17	cd11541; COG1694; pfam03819	MazG pyrophosphatase
orf07	4623–4838	+	71	8670	9.25	IME_AB3 gp10 (33%)	NC_023590	2e–04		
orf08	4822–7245	+	807	89780	5.38	vB_PaeS_SCH_Ab26 orf35 (41%)	NC_024381	0.0	COG4983	replicative primase/helicase
orf09	7593–8054	+	153	17483	8.44					
orf10	8051–8779	+	242	27281	5.42					
orf11	8845–9045	+								
orf12	9050–9307	+								
orf13	9304–9453	+								
orf14	9450–9680	+	76	8690	4.64					
orf15	9677–10063	+	128	15377	11.55					
orf16	10160–10390	+	76	8457	8.90					
orf17	10471–11160	+	229	26350	9.81					
orf18	11162–11461	+	99	11685	9.40					
orf19	11461–11901	+	146	17283	10.05					
orf20	11911–12168	+	85	9758	8.42	73 orf44 (33%)	DQ163913	0.028		
orf21	12161–12370	+	69	7604	7.34					
orf22	12367–12765	+	132	15384	6.16					
orf23	12827–12946	+	39	4365	7.15					
orf24	12947–13039	+	30	3101	8.77					
orf25	13042–13254	+	70	8056	7.19					
orf26	13256–13579	+	107	12158	9.90					
orf27	13572–13781	+	69	8124	4.89	CGphi29 orf59 (40%)	NC_020844	6e–04		
orf28	13785–14339	+	184	21030	7.89	IME_AB3 gp52 (38%)	NC_023590	8e–27		endonuclease
orf29	14375–14695	+	106	11775	8.89					
orf30	14710–14892	+	60	6533	10.39					
orf31	14889–15101	+	70	8085	9.29					
orf32	15098–15355	+	85	9657	10.40					
orf33	15352–15807	+	151	16669	7.75	TMA orf38(46%)	NC_015937	9e–29	cd01286; COG2131; pfam00383; TIGR02571; PHA02588; TIGR00326; PRK10786	deoxycytidylate deaminase
orf34	15804–16184	+	126	13761	5.97					
orf35	16246–16920	+	224	24092	5.55	IME_AB3 gp46 (35%)	NC_023590	1e–19		
orf36	17087–17476	+	129	14232	8.75	IME_AB3 gp45 (24%)	NC_023590	5e–04		
orf37	17707–18246	+	179	20145	9.34	phiHAP1 gp45 (51%)	NC_010342	2e–48	pfam05838; pfam09374; COG3926	
orf38	18247–18507	+	86	9810	10.70				PRK12792	
orf39	18681–19139	+	152	17157	5.35	73 orf5 (37%)	DQ163913	1e–15		
orf40	19117–20703	+	528	59917	5.90	KL1 orf7 (70%)	NC_018278	0.0	TIGR01547; pfam03237	Terminase large subunit
orf41	20718–22319	+	533	58562	4.77	vB_PaeKakheti25 orf8 (48%)	NC_017864	5e–151	pfam13264	Portal protein
orf42	22316–23350	+	344	38647	8.96	KL1 orf9 (28%)	NC_018278	6e–37	TIGR01641; pfam04233	Head morphogenesis protein
orf43	23373–24101	+	242	26463	5.57	vB_PaeS_SCH_Ab26 orf9 (46%)	NC_024381	2e-44	pfam14817; TIGR03345	Scaffold protein
orf44	24156–25133	+	325	35813	5.30	IME_AB3 gp37 (54%)	NC_023590	1e–115		Major capsid protein
orf45	25187–25555	+	122	13033	6.33	73 orf11 (34%)	DQ163913	3e–04		
orf46	25623–26144	+	173	18469	4.73	vB_PaeS_SCH_Ab26 orf13 (38%)	NC_024381	3e–21		Virion protein
orf47	26141–26539	+	132	14412	8.76					
orf48	26547–26999	+	150	17006	8.44	KL1 orf16 (37%)	NC_018278	6e–16		Minor tail protein
orf49	27010–28644	+	544	58390	4.52	vB_PaeKakheti25 orf17 (46%)	NC_017864	1e–128		Major tail tube protein
orf50	28731–29171	+	146	15808	5.67	IME_AB3 gp31 (36%)	NC_023590	2e–24		Tail chaperonin
orf51	29216–29494	+	92	10497	6.54	vB_PaeS_SCH_Ab26 orf18 (40%)	NC_024381	5e–13		Tail chaperonin
orf52	29491–29934	+	147	16064	9.17	73 orf19 (55%)	DQ163913	8e–42		
orf53	29939–33073	+	1044	110963	6.39	KL1 orf21 (41%)	NC_018278	3e–142	TIGR02675; pfam09718; TIGR01541; COG5281	Tail tape measure
orf54	33078–34382	+	434	49282	4.46	JWX orf22(35%)	KP202969	5e–40		
orf55	34387–34839	+	150	15660	9.04	8324 orf21 (43%)	KP202970	7e–28		
orf56	34870–35967	+	365	36841	7.31					
orf57	36005–36544	+	179	20360	5.47	JWX orf25(47%)	KP202969	1e–46	pfam08875	
orf58	36541–36936	+	131	14701	6.32	JWX orf26(50%)	KP202969	3e–38		
orf59	36933–40928	+	1331	145652	5.78	JWX orf27(42%)	KP202969	0.0	pfam09327; pfam13550; COG4733	tail component protein
orf60	40925–41347	+	140	15403	4.62					
orf61	41325–41516	+	63	7304	9.43				TIGR03798	
orf62	41560–43680	−	706	80634	7.12	IME_AB3 gp20 (55%)	NC_023590	0.0	cd05538; COG0417; TIGR00592; smart00486; PRK05761; pfam10108	DNA polymerase subunit
orf63	43680–44720	−	346	36538	7.24	vB_PmaS_IMEP1 gp03 (33%)	NC_026608	9e–53		DNA polymerase III beta subunit
orf64	44704–45045	−	100	11269	8.51	vB_PaeKakheti25 orf29 (48%)	NC_017864	9e–07		

^a^amino acids.

**Table 3:** Virion proteins detected by LC/ESI/MS/MS.

Protein ID	Score	Mass	Matches	Sequences	emPAI	Annotated Function
AKJ71391.1	139	61431	13 (6)	8 (5)	0.31	DNA/RNA helicase protein
AKJ71393.1	63	25248	3 (1)	3 (1)	0.13	recombinase
AKJ71411.1	15	15384	7 (1)	1 (1)	0.22	hypothetical protein
AKJ71414.1	15	8056	1 (1)	1 (1)	0.43	hypothetical protein
AKJ71417.1	265	21030	18 (14)	11 (9)	5.76	endonuclease
AKJ71360.1	57	24092	3 (3)	2 (2)	0.3	hypothetical protein
AKJ71365.1	140	59917	17 (11)	14 (10)	0.7	Terminase large subunit
AKJ71366.1	2203	58562	111 (73)	35 (31)	11.28	Portal protein
AKJ71369.1	27501	35813	975 (827)	32 (30)	757.42	Major capsid protein
AKJ71371.1	36	18469	1 (1)	1 (1)	0.18	Virion protein
AKJ71373.1	16	17006	3 (1)	2 (1)	0.2	Minor tail protein
AKJ71374.1	7912	58390	378 (269)	31 (31)	40.3	Major tail tube protein
AKJ71378.1	81	110963	9 (1)	8 (1)	0.03	Tail tape measure
AKJ71379.1	1146	49282	56 (44)	22 (20)	4.39	hypothetical protein
AKJ71381.1	245	36841	13 (12)	6 (6)	1.36	hypothetical protein
AKJ71382.1	30	20360	1 (1)	1 (1)	0.16	hypothetical protein
AKJ71384.1	53	145652	7 (3)	7 (3)	0.07	tail component protein
AKJ71388.1	47	36538	5 (1)	4 (1)	0.09	DNA polymerase III beta subunit
